# Estimation of Mechanical Performance, Thermal Stability and Flame Retardancy of High-Impact Polystyrene/Surface-Modified APP/Carboxylic-Functionalized MWCNTs Nanocomposites

**DOI:** 10.3390/polym11040615

**Published:** 2019-04-03

**Authors:** Li Ding, Zhimeng Jia, Hao Sun, Yong Pan, Jianping Zhao

**Affiliations:** 1 College of Mechanical and Power Engineering, Nanjing Tech University, Nanjing 211816, China; dilydily@163.com; 2 College of Safety Science and Engineering, Nanjing Tech University, Nanjing 211816, China; ars-zjia@njtech.edu.cn (Z.J.); sunever951207@outlook.com (H.S.)

**Keywords:** nanocomposites, ammonium polyphosphate, MWCNTs, high-impact polystyrene, thermal stability, flame retardancy

## Abstract

An ammonium polyphosphate (APP) surface-modified by silane coupling agent was used as flame retardant in high-impact polystyrene (HIPS). A series of HIPS nanocomposites containing different mass fractions of APP (k-APP) surface-modified by silane coupling agent (3-aminopropyl triethoxysilane, KH 550) and carboxylic-functionalized MWCNTs (COOH–MWCNTs) were prepared by the melt blending method. A composite only containing APP was also prepared as a reference material. Scanning electron microscopy (SEM) was employed to investigate the dispersion of the fillers into the HIPS matrix, and it was found the hydrophobic groups on the k-APP surface would greatly enhance the dispersion and prevent agglomerations compared with APP. Furthermore, the COOH–MWCNTs also showed good dispersibility into the matrix. Mechanical tests of the nanocomposites revealed that k-APP exhibits a more beneficial effect on both tensile and flexural properties compared with APP. Thermogravimetric analysis (TGA) and cone calorimeter tests (CCT) were conducted to probe the thermal and flammability properties of the nanocomposites, respectively. The synergistic effects of k-APP and COOH–MWCNTs on mechanical, thermal and flammability properties were examined as well.

## 1. Introduction

High-impact polystyrene (HIPS) synthesized by grafting is a widely used thermoplastic copolymer in packaging, toys, bottles, electronic appliances, light-duty industrial components, etc. due to its low cost, easy processing, and high impact strength [1]. However, the relatively poor heat deflection, high flammability and smoke production during burning limit its application [2,3]. To reduce the fire risk, some flame-retardant additives are added, such as clays, double hydroxides, phosphorous compounds, nanomaterials [4,5,6,7]. Some additives, such as ammonium polyphosphate (APP), provide the flame retardancy by promoting polymers to carbonization and forming a barrier char layer to isolate air [7]. Some other additives, such as aluminum trihydroxide (ATH) and magnesium hydroxide (MH), play the role of coolant and thinner with the aid of endothermic decomposition and release of water in the gas phase [8].

Nanomaterials have many special merits compared with normal materials, like “little size effect”, “surface effect”, “quantum effect” and “macroscopic quantum tunneling effect”. The potential use of nanomaterials in improving polymer composites’ thermal stability, flame retardancy, and mechanical performance has been demonstrated by previous works [9,10,11,12,13,14]. Carbon nanotubes (CNTs) are believed to open new avenues in the fields of polymer-based nanocomposites, considering CNTs’ huge specific surface area, outstanding thermal property, high mechanical property and low density [15,16]. It has been proved that even a small amount of CNTs could significantly reduce the peak heat release rate (PHRR) of a variety of polymers by forming a jammed network structure in the polymer matrix [11,17].

Recently, researchers have attempted to add flame retardants accompanied by nanomaterials into the polymer matrix to further improve the flame retardancy. A lesser amount of additive was used due to the nano effect of nanomaterials. Laachachi A. et al. [18,19] conducted successive studies on the effect of metal oxide nanoparticles and phosphorus flame retardant in polymethyl methacrylate (PMMA) matrix. The results revealed that both nano-alumina and nano-titanium dioxide have synergies with phosphorus flame retardant in thermal stability while only nano-alumina has a synergistic effect with phosphorus flame retardant in fire behavior of PMMA. Ye et al. [20] indicated that a suitable amount (about 2%) of multi-walled carbon nanotubes (MWCNTs) has a synergistic effect with MH in ethylene vinyl acetate copolymer (EVA) matrix in both thermal stability and flame retardancy. Cinausero N. et al. [21] indicated that hydrophobic nano-oxides have better synergistic effect with APP to improve thermal stability and fire resistance behavior compared with hydrophilic oxides, which could be attributed to the catalytic effect of hydrophobic oxides. Yen et al. [22] prepared several ethylene–propylene–diene-monomer (EPDM) rubber composites containing ATH and nano-clay. The results showed that the substitution of a part of ATH with nano-clay would enhance the thermal stability, flame retardancy, and mechanical properties of the EPDM nanocomposites. Shi et al. [23] prepared ternary polystyrene/graphitic carbon nitride/acidized multi-walled carbon nanotube (PS/g-C_3_N_4_/aMWCNT) assembled systems by the layer-by-layer assembly (LBL) method and indicated that both the thermal stability and the flame retardancy of the systems were remarkably improved compared with the PS host. Yang et al. [24] used calcium magnesium phytate (CaMg-Ph) combined with acid-treated carbon nanotubes (CNT) to prepare poly(lactic acid) (PLA) biocomposites and implied that CaMg-Ph and CNT reduces PHRR and total heat release (THR) significantly and enhanced the thermal stability of PLA biocomposites as well.

In this work, surface-modified APP (k-APP) by 3-aminopropyl triethoxysilane (KH 550) was first prepared by surface modifying APP with silane coupling agent KH-550 to improve its compatibility in HIPS matrix, and then incorporated into HIPS along with carboxylic-functionalized MWCNTs (COOH–MWCNTs). A series of HIPS/k-APP/COOH–MWCNTs nanocomposites were prepared using the melt blending method and the mechanical performance tests, thermogravimetric analysis and cone calorimeter tests were conducted. The effects of k-APP and COOH–MWCNTs on mechanical, thermal and flammability properties of the HIPS nanocomposites were investigated.

## 2. Experimental Methodology

### 2.1. Materials

**Figure 1:**
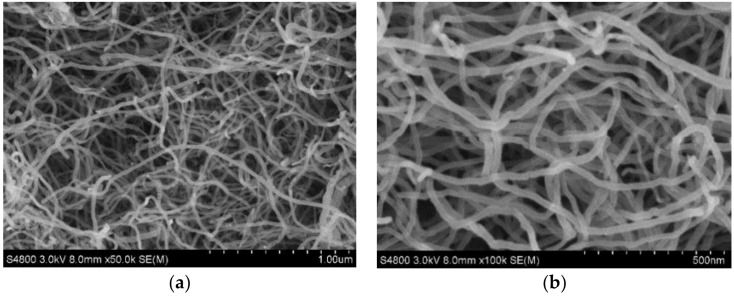
The SEM images of COOH–MWCNTs: (**a**) ×50k, (**b**) ×100k.

HIPS (PH-88, d = 1.05 g/cm) was purchased from Zhenjiang Chi Mei Chemicals Co. Ltd. (Zhenjiang, China). COOH–MWCNTs, which exhibit better dispersibility compared with virgin MWCNTs, were obtained from Chengdu Organic Chemicals Co. Ltd., Chinese Academy of Sciences (Chengdu, China) and used as-received. The basic properties of COOH–MWCNTs are listed in Table 1 and SEM images of COOH–MWCNTs provided by the vender are shown in Figure 1. APP (phase II, the degree of polymerization ≥ 1000) was purchased from Jinan Taixing Fine Chemicals Co. Ltd. (Jinan, China). Silane coupling agent KH-550 (3-aminopropyltriethoxysilane) purchased from Sinopharm Chemical Reagent Co. Ltd. (Shanghai, China) was used to surface modify APP. Analytical grade ethanol was purchased from Wuxi Yasheng Chemical Co. Ltd. (Wuxi, China).

### 2.2. Surface Modification of Ammonium Polyphosphate

APP is a hydrophilic inorganic material while the HIPS and COOH–MWCNTs are hydrophobic. Poor dispersibility and compatibility of hydrophilic material in hydrophobic material would affect the formation process and properties of composites. It has been proved that this situation can be improved by the surface modification of APP with silane coupling agent KH-550 [25,26].

To modify the surface of APP, KH-550, ethanol and distilled water in a volume ratio of 20:72:8 were mixed and then added to a three-necked flask at room temperature. The mixed solution was stirred at 50 °C in a water bath for 30 min to obtain the hydrolyzed KH-550. Thereafter, a miscible liquid of hydrolyzed KH-550 (2 g), APP (40 g) and ethanol (150 mL) was kept at 60 °C in a water bath and stirred for 1 h, and then filtered. The filter cake was washed by ethanol and deionized water several times and subsequently dried in a furnace at 60 °C for 4 h.

### 2.3. Preparation of Nanocomposites

The HIPS/k-APP/COOH–MWCNTs nanocomposites were prepared by the melt blending method after these materials were dried in an oven at 60 °C for 1 h. HIPS pellets were mixed with APP, k-APP, and COOH–MWCNTs (HIPS+APP, HIPS+k-APP, HIPS+k-APP+MWCNT, HIPS+ MWCNT) successively for about 10 min using a two-roll rubber mixing mill machine (XK-160, Shanghai first rubber machinery works, China) at 180 °C and 100 rpm. After that, hot-pressing at 180 °C under the pressure of 10 MPa was carried out for 10 min and different moulds were used to obtain 2, 3 and 4 mm sheet nanocomposites. Finally, the obtained sheets were cut into suitable size for further analysis. The mixing weight ratios of HIPS and fillers are given in Table 2.

### 2.4. Measurements and Characterization

Dispersion of the nanocomposites was examined with a JSM-7800F scanning electron microscope (SEM) (Jeol, Japan) and SmartLab X-ray diffraction patterns (XRD) (Rigaku Co., Akishima, Japan). In SEM tests, the samples were treated with spray gold after brittle fracturing in liquid nitrogen, and then their microstructures were observed. In the XRD test, the samples were recorded on an X-ray diffractometer, using a Cu Kα tube radiation (λ = 0.1542 nm) at 40 kV and 30 mA.

The tensile and flexural tests were carried out using a CMT 4254 electronic universal testing machine (Shenzhen SANS Testing Machine Co., Ltd., Shenzhen, China) at stable rates of 5 and 2 mm/min, according to ISO 527 and ISO 178 standards, respectively. Each scenario was repeated at least three times.

Thermogravimetric analysis (TGA) was conducted with an SDT Q600 DSC/TGA sync analyzer (TA Instruments, New Castle, DE, USA) operating in nitrogen atmosphere. Each test was repeated at least three times and carried out under a gas flow of 100 cm^3^/min from ambient temperature to 1000 °C at a rate of 10 °C/min with alumina crucibles (70 μL) containing 6–8 mg of sample.

The cone calorimeter tests (CCT) were performed with a CC-2 cone calorimeter (Govmark Testing Services, Inc., Farmingdale, NY, USA) to measure the flammability of HIPS and its nanocomposites according to ISO5660. Each sample with a dimension of 100 × 100 × 3 mm^3^ was exposed horizontally to the conic heat radiator with an external heat flux of 50 kW/m^2^. Each test was repeated at least three times.

## 3. Results and Discussion

### 3.1. Surface Modification Treatment and Thermal Stability Characterization of APP

**Figure 2:**
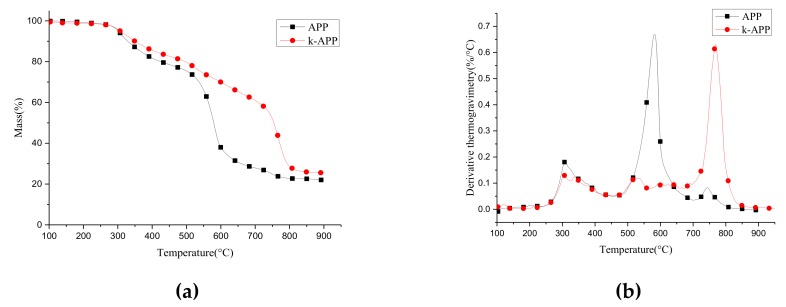
TG (**a**) and DTG (**b**) curves of APP and k-APP (in N_2_).

The modification method with a coupling agent has been widely used in the modification of inorganic fillers in the preparation of polymer composites due to its simple operation and good reliability. It is necessary to compare the thermal stability between APP and k-APP [27]. Figure 2 shows the thermogravimetric (TG) and derivative thermogravimetry (DTG) curves of APP and k-APP in N_2_. The thermal decomposition process of APP can be divided into three stages: 250–450 °C, 450–700 °C and above 700 °C. As temperature increases in the first stage, unstable groups such as amino in APP decompose and release NH_3_ and small amount of H_2_O. During the second stage, higher temperature causes thermal decomposition of the phosphoric acid, which releases NH_3_ and considerable H_2_O and forms phosphoric acid, pyrophosphate, polyphosphoric acid and crosslinked P_2_O_5_ [28]. In the last stage, the previously generated phosphoric acid, pyrophosphate, polyphosphoric acid etc. are decomposed, leaving about 21% residue of the original APP mass.

Obviously, the thermal stability of k-APP differs a lot compared with APP, which decomposes significantly as the temperature rises up to 280 °C. Specifically, the thermal decomposition mass loss of k-APP is slightly lower than that of APP between 250–450 °C and it is only about 21% between 450 and 700 °C. On the contrary, about 50% mass loss of APP occurs in this temperature range. When the temperature rises higher than 700 °C, the third step thermal decomposition of k-APP occurs, and the mass loss in this stage is as high as 35%. It is probably because the number of –OH directly connected to the P atom in k-APP is reduced owing to the coupling reaction, and the Si–O–P structure with higher bond energy is formed, which improves the activation energy for thermal decomposition of the super phosphoric acid and postpones the cross-linking reaction. Therefore, k-APP was used to prepare the desired nanocomposites (HIPS+k-APP, HIPS+k-APP+MWCNT) in the following experiments.

### 3.2. Dispersion of the Fillers

**Figure 3:**
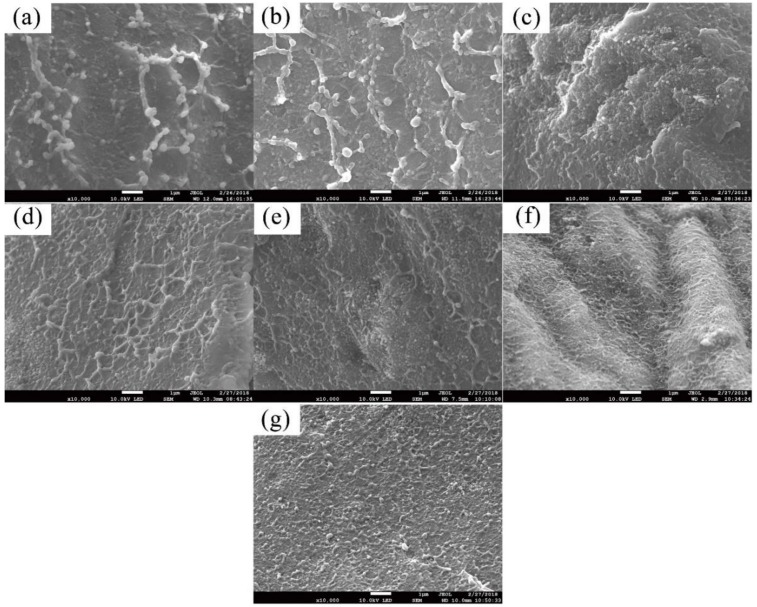
SEM images of a section of the HIPS nanocomposites (×10k): (**a**) HIPS1, (**b**) HIPS2, (**c**) HIPS3, (**d**) HIPS4, (**e**) HIPS5, (**f**) HIPS6, and (**g**) HIPS7.

In order to better understand the flame-retardant mechanism [29], both SEM and XRD experiments were carried out in this study. The SEM micrographs for the brittle fracture section of all the samples except HIPS0 are shown in Figure 3, which confirms the effect of the coupling agent. Figure 3b,c shows that the k-APP exhibits better dispersion into the HIPS matrix and fewer agglomerations compared with APP, mainly owing to the treatment of the coupling agent. COOH–MWCNTs could also be dispersed uniformly into the matrix, attributed to the presence of hydrophilicity carboxyl groups on the surface irrespective of whether they were added separately or blended with k-APP in the matrix.

**Figure 4:**
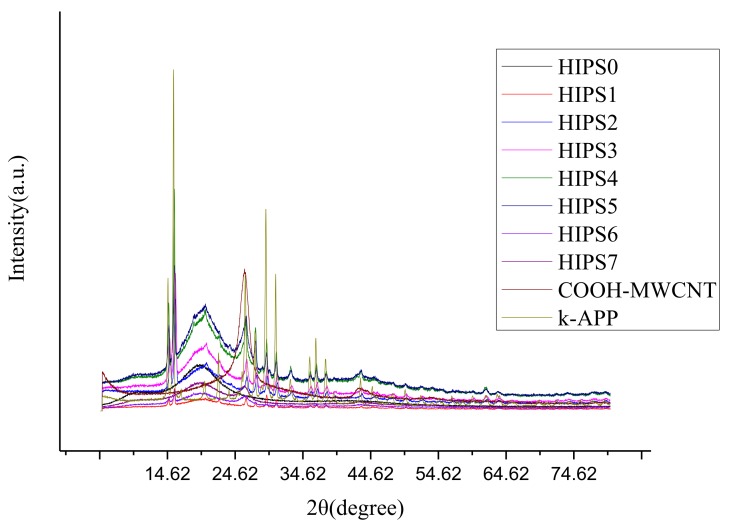
XRD patterns of HIPS0, HIPS1, HIPS2, HIPS3, HIPS4, HIPS5, HIPS6, HIPS7, COOH–MWCNT and k-APP.

Figure 4 presents XRD patterns of HIPS0, HIPS1, HIPS2, HIPS3, HIPS4, HIPS5, HIPS6, HIPS7, COOH–MWCNT and k-APP. Apparently, all the nanocomposites have a broad peak at about 20°, which results from the amorphous feature of the cross-linked polymer networks [30]. Besides, the peaks of HIPS2, HIPS3, HIPS4, HIPS5, HIPS5 are similar to the peaks of k-APP at about 15°, 27°, 30°, 31°, 37°, 38°. Analogously, the peaks of HIPS4, HIPS5, HIPS5, HIPS6, HIPS7 are similar to the peaks of COOH–MWCNT at about 26°. The results indicate that k-APP and COOH–MWCNTs are dispersed uniformly into the HIPS matrix, which coincides with the results of SEM images mentioned above.

### 3.3. Mechanical Performance

**Figure 5:**
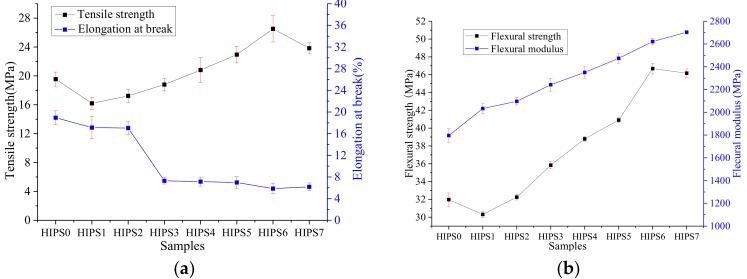
Mechanical properties of HIPS and its nanocomposites: (**a**) tensile strength and elongation at break; (**b**) flexural strength and modulus.

Mechanical properties of polymers are very important to their applications in engineering. Figure 5 shows the mechanical properties of HIPS and its nanocomposites, including the tensile and flexural properties. Adding k-APP and COOH–MWCNTs into the HIPS matrix has a more significant reinforced effect on the flexural property than on tensile property. The variation tendencies for tensile and flexural strengths are similar; both decrease first after the addition of APP, and then increase obviously because all APPs are replaced by k-APPs. The tendencies mainly result from the good dispersibility and interfaces compatibility of k-APP in the HIPS matrix attributed to the treatment by the silane coupling agent KH-550, which makes the k-APP particles uniformly embedded in the polymer matrix and further improves the tensile and flexural strengths of the nanocomposites compared with k-APP, as supported by Lin et al. [25]. Based on this, further improvement is observed when gradually replacing k-APP with COOH–MWCNTs. This result may be interpreted by two reasons. First is the graft of –COOH on the MWCNTs surface can produce good interface interaction between the COOH–MWCNTs and the HIPS matrix, which promotes its uniform dispersion into the matrix and transfers the external load to COOH–MWCNTs [31]. The second is the COOH–MWCNTs in the matrix can transfer the load by virtue of their own high length-diameter ratio and large specific surface area, and improve the tensile strength of the nanocomposites by absorbing a part of the energy due to their high modulus and strength. It is worth noting that both the tensile and flexural strengths of HIPS6 are higher than those of HIPS7, which contains 15 wt % COOH–MWCNTs. It is inferred that there is synergistic effect on both tensile and flexural strengths when the nanocomposites contain 3 wt % k-APP and 12 wt % COOH–MWCNTs.

Figure 5a shows the elongation at the breakage of HIPS and its nanocomposites. It indicates that APP can reduce the elongation at breakage of the nanocomposites slightly, even though the surface-modified APP by the silane-coupling agent cannot improve it. In addition, the elongation at breakage of the nanocomposites would be greatly reduced by replacing a part of k-APP in the HIPS/k-APP composite with COOH–MWCNTs, but it is barely affected by the alteration of the COOH–MWCNTs content.

In Figure 5b, the k-APP better enhances flexural modulus of the nanocomposites compared with APP. Consequently, the flexural modulus of the HIPS nanocomposites, which replaced a part or all of k-APP by COOH–MWCNTs, is further enhanced by the weight fraction of COOH–MWCNTs increasing from 3 to 15 wt %. When the weight fraction of COOH–MWCNTs is 15 wt %, over 50% growth of flexural modulus is observed, compared with pure HIPS. The effectiveness of load transfer among the nanotube fillers, the nanotube—matrix interfacial strength and the general orientation of the nanotubes in the matrix is considered to be the factor that mainly influences the modulus in nanotube composites [32]. Considering the random orientation distribution of COOH–MWCNTs in the HIPS matrix, the main cause of the modulus increment may be the effectiveness of load transfer between the nanotubes and the nanotube—matrix interfacial strength, which seems to be improved by the good dispersibility and network structure of COOH–MWCNTs in the HIPS matrix as shown in Figure 3.

### 3.4. Thermogravimetric Analysis

**Figure 6:**
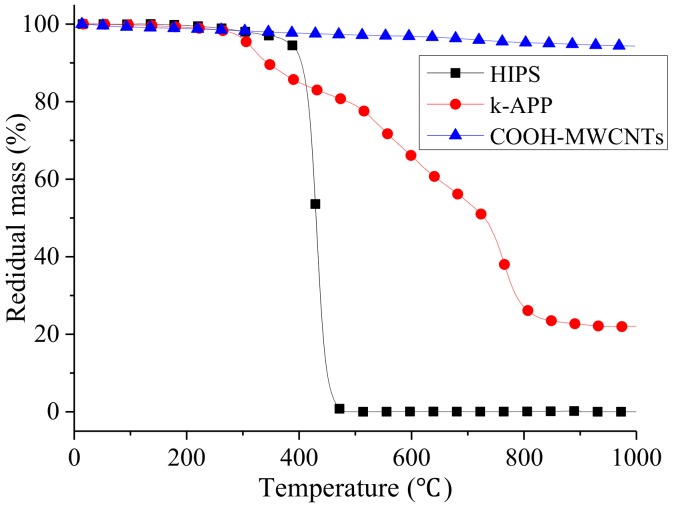
TG curves of HIPS, k-APP and COOH–MWCNTs in the nitrogen atmosphere.

Thermal stability of polymers can be used to characterize both their thermal degradation and aging resistance. Figure 6 presents the thermogravimetric (TG) curves from ambient temperature to 1000 °C obtained at 10 °C/min for HIPS, k-APP, and COOH–MWCNTs in N_2_. Distinctly, pure HIPS undergoes a single thermal decomposition reaction in the temperature range of 283–360 °C while k-APP decomposes through three reactions in the temperature ranges of 283–360 °C, 507–606 °C, and 737–798 °C, respectively. Moreover, COOH–MWCNTs show excellent thermal stability with more than 94 wt % residue left after being heated to 1000 °C. A slight mass loss of about 6 wt % is probably related to the low thermal stability oxygen-containing groups existing on the surface and end of COOH–MWCNTs, such as -COOH, –C=O, and –OH [33].

**Figure 7:**
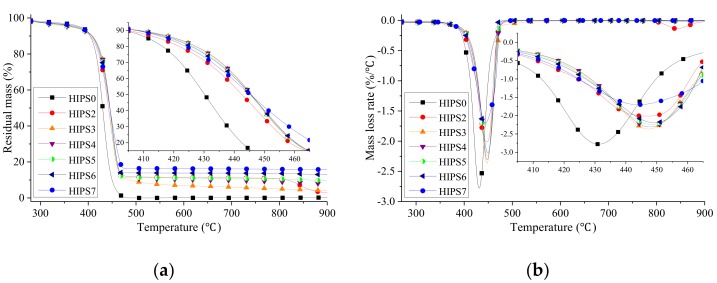
TG (**a**) and DTG (**b**) curves of HIPS and its nanocomposites in nitrogen atmosphere.

The TG and DTG curves of pure HIPS and its nanocomposites individually containing k-APP and COOH–MWCNTs or in combination from 300 to 900 °C under N_2_ atmosphere are shown in Figure 7. Also, the critical data of thermal analysis for the HIPS nanocomposites, including the onset decomposition temperature (*T_d, onset_*), the temperature at maximum mass loss rate (*T_max_*), the peak mass loss rate (PMLR) and the residue yield, are summarized in Table 3. It can be found that all samples show a single step thermal decomposition reaction except HIPS2, which has a negligible degradation reaction in the temperature range of 352–372 °C and losses about 20% weight of k-APP (namely, 3 wt % of HIPS2). It is considered that the dominant mechanisms of the single degradation step of pure HIPS are depolymerization and cyclization [34] with hardly any residue left. Therefore, the two mass loss steps of HIPS2 are related to the first and third degradation steps of k-APP, which seem to be delayed about 60 and 80 °C, respectively, when APP is added. However, the mass loss step over 800 °C does not exist in the thermal degradation process of the nanocomposites which contain both k-APP and COOH–MWCNTs. The possible reason is the different effects of k-APP and COOH–MWCNTs in HIPS. Czegeny et al. [35] explained the alteration of the decomposition mechanism of polystyrene which is abundant in HIPS with the presence of APP. The effect of k-APP mainly exists on two sides. The interaction between the phenyl side group and polyphosphoric acid, the product of the second decomposition of k-APP, can reduce the electron donating ability of the phenyl side group. The interaction reduces the macromolecular radical reaction rate of the phenyl side group and inhibits the backbiting of PS. Moreover, cyclisation reactions occur resulting from the interaction between radical sites of polystyrene and ionic species of k-APP, further promoting the formation of char. On the contrary, as the inert filler in PS or HIPS, MWCNTs have no marked effect on the decomposition mechanism of the polymer matrix. Armed with uniform dispersion and network structure formation, MWCNTs may help provide a physical barrier against the permeation of volatiles and smoke particles [36]. In Figure 7, the temperature range of the first step thermal decomposition of k-APP is close to the main mass loss temperature range. It indicates that COOH–MWCNTs could delay or block the chain reaction of the first step thermal decomposition of k-APP by virtue of its strong free radical capture ability at the initial stage of thermal decomposition. As a result, the aforementioned negligible degradation step does not exist in all of the HIPS/k-APP/COOH–MWCNTs nanocomposites samples. After the free radical capture ability of COOH–MWCNTs reached its limit, the third segment mass loss of k-APP is accelerated and advanced to occur together with the single step thermal decomposition of the HIPS matrix probably due to the potential catalytic activity of MWCNTs and the high reaction activity of carboxyl group (–COOH), which leads to only one step of mass loss for all HIPS/k-APP/COOH–MWCNT nanocomposites. Additionally, it could be observed from Figure 7a and Table 3 that the residue yields of HIPS2, HIPS3, HIPS4, HIPS5, HIPS6, and HIPS7 at 900 °C are 2.90%, 4.13%, 7.81%, 9.68%, 12.92% and 15.76%, respectively. All the results are originated from the promotion effect of k-APP and its thermal degraded products on the formation of char [37] and from the excellent thermal stability of COOH–MWCNTs.

The data in Table 3 indicate that the individual presence of k-APP and COOH–MWCNTs or combination exhibits an apparent raise in *T_d,onset_* and *T_max_* of the HIPS nanocomposites. Concretely speaking, 15 wt % more additives result in the delays of nanocomposites *T_d,onset_* by 5.55 (15% k-APP), 12.42 (12% k-APP + 3% COOH–MWCNTs), 12.25 (9% k-APP + 6% COOH–MWCNTs), 11.13 (6% k-APP + 9% COOH–MWCNTs), 10.46 (3% k-APP + 12% COOH–MWCNTs), and 3.53 °C (15% COOH–MWCNTs) compared with pure HIPS, while the delays of *T_max_* are 15.67, 16.24, 16.73, 17.12, 18.15, and 13.06 °C, respectively. Mass loss of k-APP due to its first degradation step seems to have no negative impact on *T_d,onset_* of the HIPS nanocomposites mainly due to the slight mass loss. These results reveal the existence of a synergistic effect between k-APP and MWCNTs in improving the thermal stability of HIPS. Moreover, the measured values of PMLR are significantly reduced due to the separate addition of k-APP and COOH–MWCNTs or combination. However, no further reduction of PMLR is found when both additives are used. Also in Table 3, the variation trends of *T_d,onset_* and *T_max_* for HIPS3, HIPS4, HIPS5, and HIPS6 are opposite. Both results could be attributed to the different effects of two additives and their interaction in the HIPS matrix. The release of ammonia and water, gaseous products of the degradation of k-APP, helps retard the degradation process of HIPS via diluting the radicals, resulting in the delays of *T*_d,onset_ of the HIPS nanocomposites. Thus, the yield of the gas product reduces gradually with the increasing content of k-APP substituted by COOH–MWCNTs leading to its attenuated dilution effect and the shifted *T_d,onset_* of the HIPS nanocomposites toward lower temperature. Additionally, considering the existence of COOH–MWCNTs possibly catalyzing the third mass loss step of k-APP, the reduction of k-APP would increase the *T_max_* and reduce the PMLR of the HIPS/k-APP/COOH–MWCNTs nanocomposites.

### 3.5. Cone Calorimeter Test Analysis

The interaction between the two additives in the matrix during the combustion process is investigated via cone calorimeter test (CCT). The obtained flammability properties of pure HIPS and its nanocomposites such as TTI (time to ignite), PHRR (peak heat release rate), THR (total heat release), CO yield, CO_2_ yield and residue yield are listed in Table 4. Irrespective of whether the two additives are incorporated individually or together, the TTIs of the nanocomposites are reduced compared with pure HIPS. It is mainly because pure HIPS and other carbon-based polymers have quite obvious transmission bands which can absorb the external radiation heat in a large depth range, while the additives would cause the composite material to lose the transmission band and the thermal radiation to be concentrated on its surface [38].

**Figure 8:**
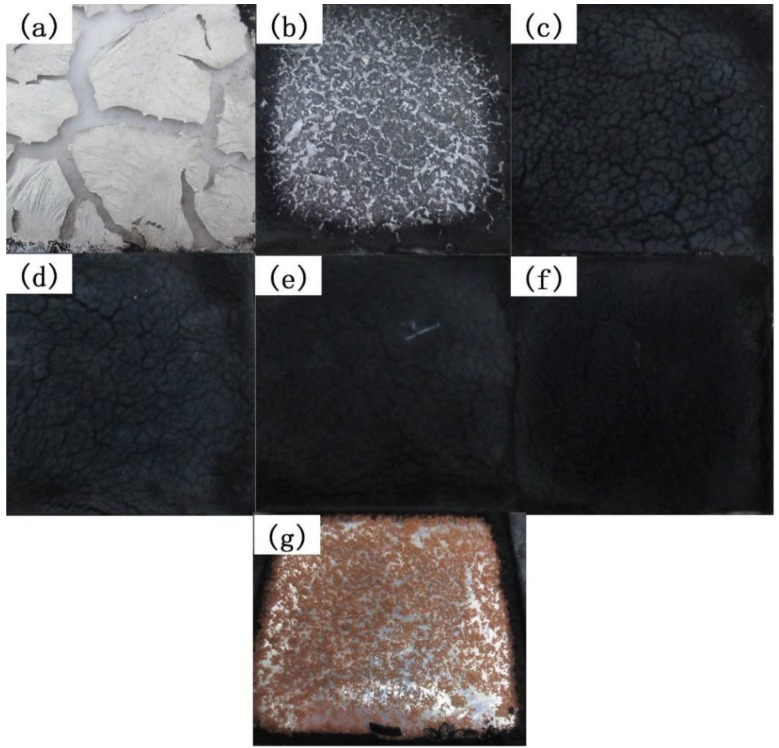
Digital photographs for residues of pure HIPS and its nanocomposites after CCT: (**a**) HIPS0; (**b**) HIPS2; (**c**) HIPS3; (**d**) HIPS4; (**e**) HIPS5; (**f**) HIPS6; (**g**) HIPS7.

Figure 8 shows the digital photographs of residuals of pure HIPS and its nanocomposites after CCT. The simultaneous existences of k-APP and COOH–MWCNTs are able to form the compact char layers as shown in Figure 8c–f, and the compactness of char layer increases with the increasing carbon nanotubes content in the nanocomposites. A large number of orange/red residues are found instead of the black char layer after CCT for HIPS6 as shown in Figure 8g. The reason has been proved to be Fe_2_O_3_ formed by the oxidation of Fe catalyst in the process of preparing CNTs, which was confirmed by Kashiwagi et al. [38].

**Figure 9:**
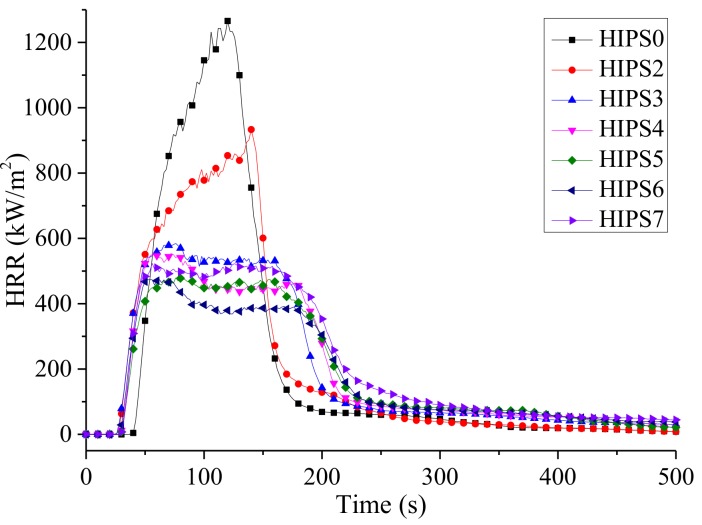
HRR curves of HIPS and its nanocomposites.

**Figure 10:**
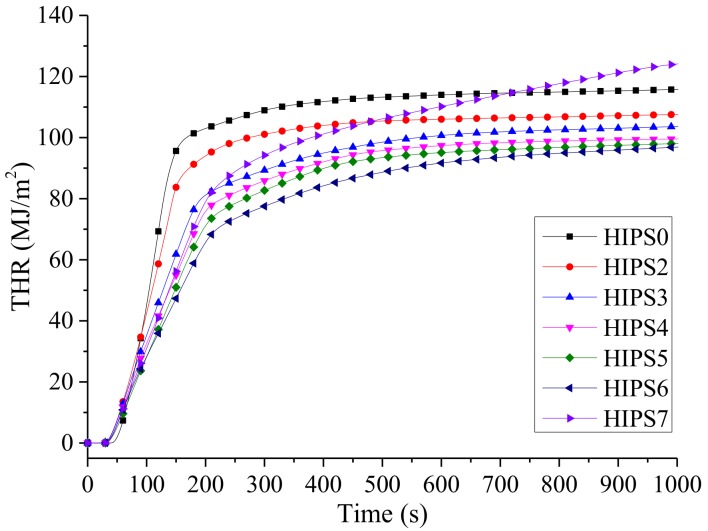
THR curves of HIPS and its nanocomposites.

Figure 9 and Figure 10 are the curves for HRR and THR of HIPS and its nanocomposites, respectively. Combining Table 4, it can be found adding 15 wt % k-APP to the HIPS matrix can reduce the PHRR and THR of the nanocomposites by about 26.27% and 8.26%, respectively. The gaseous thermal decomposition products of k-APP, such as NH_3_ and H_2_O, show a dilution effect on the gaseous small molecular products generated by the thermal decomposition of HIPS. Moreover, phosphoric acid is also generated during the thermal decomposition of k-APP. The combination of the foaming effect of gaseous products mentioned above and the cross-linking effect of phosphoric acid can also form a char layer leading to barrier effect. This char layer can hinder heat conduction and volatilization of HIPS thermal decomposition products, and greatly slows down the thermal decomposition. Furthermore, the growth of HRR would be hindered, and eventually the PHRR and THR of the HIPS/k-APP/COOH–MWCNTs nanocomposites would be decreased. Increasing the content of COOH–MWCNTs in the HIPS nanocomposites can reduce the PHRR and THR of the nanocomposites gradually and prolong the combustion process. The reasons attributed to the barrier effect of the compact char layer formed by COOH–MWCNTs and k-APP on the volatilization of gaseous thermal decomposition products of HIPS and heat transfer. The related data in Table 3 indicate that k-APP and COOH–MWCNTs play a synergistic role in reducing the PHRR and THR of the HIPS nanocomposites and the synergistic effect is enhanced with the increasing COOH–MWCNT content.

**Figure 11:**
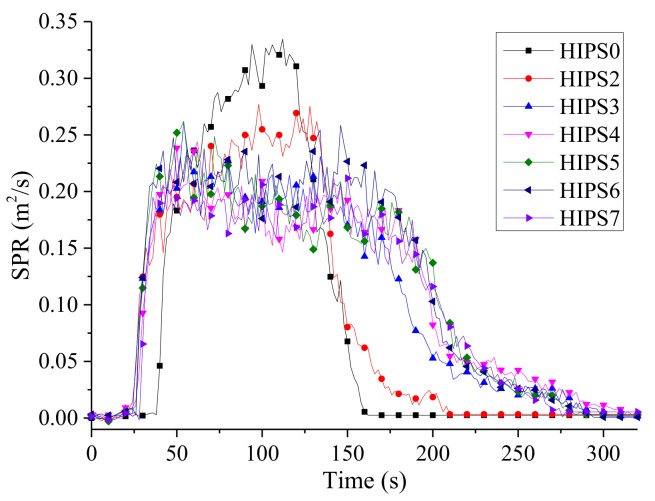
SPR curves of HIPS and its nanocomposites.

The SPR (smoke production rate) curves of HIPS and its nanocomposites are shown in Figure 11. The peak values of SPR for the nanocomposites are significantly reduced whether the k-APP and COOH–MWCNTs are added individually or in combination. When COOH–MWCNTs are present in the nanocomposites, the smoke release process is prolonged due to the barrier effect provided by the compact char layer mentioned above. Compared with pure HIPS, it can be seen in Table 4 that both the CO and CO_2_ yields of the HIPS nanocomposites are significantly decreased as the fillers are added. It is worth noting that the CO and CO_2_ yields of HIPS3, HIPS4, HIPS5, and HIPS6 are higher than those of HIPS2 and HIPS7. The CO yield increases with the increasing COOH–MWCNTs content in the nanocomposites, while the change trend of CO_2_ yield is the opposite. The increase in CO yield is mainly due to incomplete combustion of the nanocomposites resulting from the barrier effect of the compact and stable char layer formed by k-APP and COOH–MWCNTs. There is a positive correlation between the density of the char layer and the content of COOH–MWCNTs in the matrix as shown in Figure 8. Therefore, the CO yield of the sample increases with the increasing COOH–MWCNT content.

**Figure 12:**
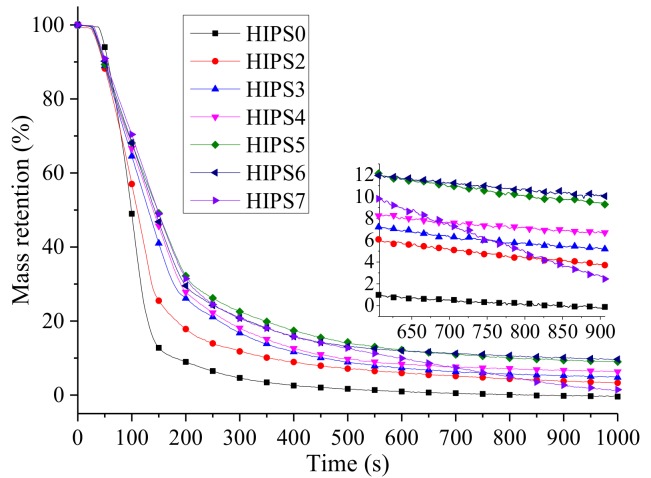
MLR curves of HIPS and its nanocomposites.

The variation curves of mass for HIPS and its nanocomposites are shown in Figure 12. Mass loss of the HIPS nanocomposites occurs earlier than that of pure HIPS owing to the lower TTI value. However, the mass loss rate of the nanocomposites is significantly lower than that of pure HIPS. Furthermore, the residues of HIPS0, HIPS2, HIPS3, HIPS4, HIPS5, HIPS6, and HIPS7 are 0%, 3.04%, 4.59%, 6.42%, 8.24%, 8.51% and 1.04%, respectively. It can be conclude that k-APP and COOH–MWCNTs have a synergistic effect on increasing the residual amount of the HIPS nanocomposites and the k-APP plays a more important role in promoting the char layer formation than COOH–MWCNTs. It can be observed from Figure 10 and Figure 12 that the weight of HIPS7 is lost constantly and the heat is released continuously during the tests. These results show that the stability of the char layer formed by COOH–MWCNTs is worse than that formed by combination of k-APP and COOH–MWCNTs, which is another cause intensifying incomplete combustion of the HIPS/k-APP/COOH–MWCNTs nanocomposites and leading to more CO yield compared with the HIPS/k-APP nanocomposites and the HIPS/COOH–MWCNTs nanocomposites.

**Figure 13:**
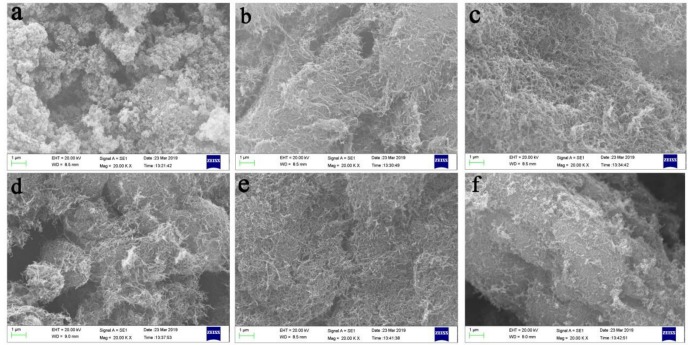
SEM images of char residue of the HIPS nanocomposites (×20k): (**a**) HIPS0, (**b**) HIPS3, (**c**) HIPS4, (**d**) HIPS5, (**e**) HIPS6, and (**f**) HIPS7.

To further understand the flame retardant mechanism, SEM experiments were also employed to investigate the char residue of HIPS0, HIPS3, HIPS4, HIPS5, HIPS6 and HIPS7 with EVO18 SEM (Zeiss, Germany). All the SEM images of char residue of the HIPS nanocomposites are shown in Figure 13. Figure 13b–f shows that dense reticular char layers exist in HIPS3, HIPS4, HIPS5, HIPS6 and HIPS7. The char layers can act as a barrier to hinder both heat transfer and volatilization of thermal decomposition products of nanocomposites, which can improve the flame retardancy and thermal stability of nanocomposites.

## 4. Conclusions

In this work, a series of the HIPS nanocomposites containing COOH–MWCNTs and k-APP prepared by surface modifying APP with silane coupling agent KH-550 individually or jointly were prepared. The dispersion of the fillers into the nanocomposites was characterized, and the mechanical, thermal, and flammability properties were explored. Although the elongation at breakage decreased, the other mechanical properties of HIPS/k-APP/COOH–MWCNTs nanocomposites increased with increasing COOH–MWCNTs content. Moreover, the chain reaction of the first step thermal decomposition of k-APP was found to be delayed or blocked by COOH–MWCNTs, whereas the third step thermal decomposition of k-APP was preceded owing to the existence of COOH–MWCNTs. It was also shown that k-APP and COOH–MWCNTs played synergistic roles in reducing the PHRR and THR of HIPS nanocomposites, and the synergistic effect was enhanced with the increasing COOH–MWCNTs content. Furthermore, the stability of the char layer formed by the COOH–MWCNTs was found to be related to the amount of k-APP added in nanocomposites. It can be deduced that the combined addition of k-APP and COOH–MWCNTs could strengthen the incomplete combustion and increase the yield of CO.

## Figures and Tables

**Table 1:** Properties of Carboxylic-functionalized Multi-Wall Carbon Nanotubes.

Designation	–COOH Content (wt %)	Diameter		L (μm)	True Density (g/cm)	Special Surface Area (m^2^/g)
		Outer (nm)	Inner (nm)			
COOH–MWCNTs	1.55	10–30	5–10	10–30	2.1	>110

**Table 2:** Formulations of HIPS and its nanocomposites.

Samples	HIPS	APP	k-APP	COOH–MWCNTS
HIPS0	100	0	0	0
HIPS1	85	15	0	0
HIPS2	85	0	15	0
HIPS3	85	0	12	3
HIPS4	85	0	9	6
HIPS5	85	0	6	9
HIPS6	85	0	3	12
HIPS7	85	0	0	15

**Table 3:** Critical data of thermal analysis for HIPS and its nanocomposites.

Samples	*T*_d, onset_ (°C)	*T*_max_ (°C)	PMLR (%/min)	Residue Yield at 900 °C (%)
HIPS0	411.87	431.10	27.79	0.00
HIPS2	417.42	446.77	20.13	2.90
HIPS3	424.29	447.34	23.67	4.13
HIPS4	424.12	447.83	23.10	7.81
HIPS5	423.00	448.22	22.40	9.68
HIPS6	422.33	449.25	21.90	12.92
HIPS7	415.40	444.16	17.03	15.76

**Table 4:** Critical data of CCT for HIPS and its nanocomposites.

Samples	TTI (s)	PHRR (kW/m^2^)	THR (MJ/m^2^)	CO Yield (kg/kg)	CO_2_ Yield (kg/kg)	Residue Yield (%)
HIPS0	37	1265.70	117.38	0.72	15.84	0
HIPS2	24	933.20	107.72	0.40	6.50	3.03
HIPS3	24	584.50	104.07	0.42	4.78	4.59
HIPS4	26	554.10	99.61	0.44	4.52	6.42
HIPS5	25	479.40	99.34	0.50	4.32	8.24
HIPS6	25	474.68	98.98	0.51	4.30	8.51
HIPS7	29	523.55	126.28	0.23	4.24	1.04

## References

[1] Chang S.Q., Xie T.X., Yang G.S. (2006). Effects of polystyrene-encapsulated magnesium hydroxide on rheological and flame-retarding properties of hips composites. Polym. Degrad. Stab..

[2] Yang Z., Zhou C.G., Yang H., Cai T., Cai J., Li H.B., Zhou D., Chen B.F., Li A.M., Cheng R.S. (2012). Improvement of the compatibilization of high-impact polystyrene/magnesium hydroxide composites with partially sulfonated polystyrene as macromolecular compatibilizers. Ind. Eng. Chem. Res..

[3] Wang B.B., Zhang Y., Tao Y.J., Zhou X., Song L., Jie G.X., Hu Y. (2018). Monitoring the degradation of physical properties and fire hazards of high-impact polystyrene composite with different ageing time in natural environments. J. Hazard. Mater..

[4] Hu Y.P., Wang X.M., Li J. (2016). Regulating effect of exfoliated clay on intumescent char structure and flame retardancy of polypropylene composites. Ind. Eng. Chem. Res..

[5] Laoutid F., Bonnaud L., Alexandre M., Lopez-Cuesta J.M., Dubois P. (2009). New prospects in flame retardant polymer materials: From fundamentals to nanocomposites. Mater. Sci. Eng. R.

[6] Braun U., Schartel B. (2004). Flame retardant mechanisms of red phosphorus and magnesium hydroxide in high impact polystyrene. Macromol. Chem. Phys..

[7] Lim K.S., Bee S.T., Sin L.T., Tee T.T., Ratnam C.T., Hui D., Rahmat A.R. (2016). A review of application of ammonium polyphosphate as intumescent flame retardant in thermoplastic composites. Compos. Part B.

[8] Hoffendahl C., Duquesne S., Fontaine G., Taschner F., Mezger M., Bourbigot S. (2015). Decomposition mechanism of fire retarded ethylene vinyl acetate elastomer (EVA) containing aluminum trihydroxide and melamine. Polym. Degrad. Stab..

[9] Lebaron P.C., Wang Z., Pinnavaia T.J. (1999). Polymer-layered silicate nanocomposites: An overview. Appl. Clay Sci..

[10] Leroux F., Besse J.P. (2001). Polymer interleaved layered double hydroxide:  a new emerging class of nanocomposites. Chem. Mater..

[11] Kashiwagi T., Du F.M., Douglas J.F., Winey K.I., Harris R.H., Shields J.R. (2005). Nanoparticle networks reduce the flammability of polymer nanocomposites. Nat. Mater..

[12] Morgan A.B. (2006). Flame retarded polymer layered silicate nanocomposites: A review of commercial and open literature systems. Polym. Adv. Technol..

[13] Coleman J.N., Khan U., Blau W.J., Gun’ko Y.K. (2006). Small but strong: A review of the mechanical properties of carbon nanotube–polymer composites. Carbon.

[14] Chattopadhyay D.K., Webster D.C. (2009). Thermal stability and flame retardancy of polyurethanes. Prog. Polym. Sci..

[15] Kashiwagi T., Du F.M., Winey K.I., Groth K.M., Shields J.R., Bellayer S.P., Kim H., Douglas J.F. (2005). Flammability properties of polymer nanocomposites with single-walled carbon nanotubes: Effects of nanotube dispersion and concentration. Polymer.

[16] Rybiński P., Anyszka R., Imiela M., Sicinski M., Gozdek T. (2017). Effect of modified graphene and carbon nanotubes on the thermal properties and flammability of elastomeric materials. J. Therm. Anal. Calorim..

[17] Durkin D.P., Gallagher M.J., Frank B.P., Knowlton E.D., Trulove P.C., Fairbrother D.H., Fox D.M. (2017). Phosphorus-functionalized multi-wall carbon nanotubes as flame-retardant additives for polystyrene and poly(methyl methacrylate). J. Therm. Anal. Calorim..

[18] Laachachi A., Cochez M., Leroy E., Gaudon P., Ferriol M., Lopez-Cuesta J.M. (2010). Effect of Al_2_O_3_ and TiO_2_ nanoparticles and APP on thermal stability and flame retardance of PMMA. Polym. Adv. Technol..

[19] Laachachi A., Cochez M., Leroy E., Ferriol M., Lopez-Cuesta J.M. (2007). Fire retardant systems in poly(methyl methacrylate): Interactions between metal oxide nanoparticles and phosphinates. Polym. Degrad. Stab..

[20] Ye L., Wu Q.H., Qu B.J. (2009). Synergistic effects and mechanism of multiwalled carbon nanotubes with magnesium hydroxide in halogen-free flame retardant EVA/MH/MWNT nanocomposites. Polym. Degrad. Stab..

[21] Cinausero N., Azema N., Lopez-Cuesta J.M., Cochez M., Ferriol M. (2011). Synergistic effect between hydrophobic oxide nanoparticles and ammonium polyphosphate on fire properties of poly(methyl methacrylate) and polystyrene. Polym. Degrad. Stab..

[22] Yen Y.Y., Wang H.T., Guo W.J. (2013). Synergistic effect of aluminum hydroxide and nanoclay on flame retardancy and mechanical properties of EPDM composites. J. Appl. Polym. Sci..

[23] Shi Y.Q., Long Z., Yu B., Zhou K., Gui Z., Yuen R.K.K., Hu Y. (2015). Tunable thermal, flame retardant and toxic effluent suppression properties of polystyrene based on alternating graphitic carbon nitride and multi-walled carbon nanotubes. J. Mater. Chem. A.

[24] Yang W., Tawiah B., Yu C., Qian Y.F., Wang L.L., Yuen A.C.Y., Zhu S.E., Hu E.Z., Chen T.B.Y., Yu B. (2018). Manufacturing, mechanical and flame retardant properties of poly(lactic acid) biocomposites based on calcium magnesium phytate and carbon nanotubes. Compos. Part A.

[25] Lin H.J., Yan H., Liu B., Wei L.Q., Xu B.S. (2011). The influence of KH-550 on properties of ammonium polyphosphate and polypropylene flame retardant composites. Polym. Degrad. Stab..

[26] Liu J.C., Xu M.J., Lai T., Li B. (2015). Effect of surface-modified ammonium polyphosphate with KH550 and silicon resin on the flame retardancy, water resistance, mechanical and thermal properties of intumescent flame retardant polypropylene. Ind. Eng. Chem. Res..

[27] Wang B.B., Qian X.D., Shi Y.Q., Yu B., Hong N.N., Song L., Hu Y. (2015). Cyclodextrin microencapsulated ammonium polyphosphate: Preparation and its performance on the thermal, flame retardancy and mechanical properties of ethylene vinyl acetate copolymer. Compos. Part B Eng..

[28] Bourbigot S., Le Bras M., Gengembre L., Delobel R. (1994). XPS study of an intumescent coating application to the ammonium polyphosphate/pentaerythritol fire-retardant system. Appl. Surf. Sci..

[29] Shi Y.Q., Yu B., Duan L.J., Gui Z., Wang B.B., Hu Y., Yuen R.K.K. (2017). Graphitic carbon nitride/phosphorus-rich aluminum phosphinates hybrids as smoke suppressants and flame retardants for polystyrene. J. Hazard. Mater..

[30] Yu B., Shi Y.Q., Yuan B.H., Qiu S.L., Xing W.Y., Hu W.Z., Song L., Lo S.M., Hu Y. (2015). Enhanced thermal and flame retardant properties of flame-retardant-wrapped graphene/epoxy resin nanocomposites. J. Mater. Chem. A.

[31] Jia Z.J., Wang Z.Y., Xu C.L., Liang J., Wei B.Q., Wu D.H., Zhu S.W. (1999). Study on poly(methyl methacrylate)/carbon nanotube composites. Mater. Sci. Eng. A.

[32] Ayewah D.O.O., Davis D.C., Krishnamoorti R., Lagoudas D.C., Sue H.J., Willson M. (2010). A surfactant dispersed SWCNT-polystyrene composite characterized for electrical and mechanical properties. Compos. Part A.

[33] Rao A.M., Richter E., Bandow S., Chase B., Eklund P.C., Williams K.A., Fang S., Subbaswamy K.R., Menon M., Thess A. (1997). Diameter-selective raman scattering from vibrational modes in carbon nanotubes. Science.

[34] Wang Y., Zhang J. (2013). Thermal stabilities of drops of burning thermoplastics under the UL 94 vertical test conditions. J. Hazard. Mater..

[35] Czégény Z., Blazsó M. (2008). Effect of phosphorous flame retardants on the thermal decomposition of vinyl polymers and copolymers. J. Anal. Appl. Pyrolysis.

[36] Sun G.X., Chen G.M., Liu Z.P., Chen M. (2010). Preparation, crystallization, electrical conductivity and thermal stability of syndiotactic polystyrene/carbon nanotube composites. Carbon.

[37] Zhu X.S. (1996). Thermal and thermo-oxidative degradation of polystyrene with ammonium polyphosphate. J. Fire Sci..

[38] Kashiwagi T., Grulke E., Hilding J., Groth K., Harris R., Butler K., Shields J., Kharchenko S., Douglas J. (2004). Thermal and flammability properties of polypropylene/carbon nanotube nanocomposites. Polymer.

